# MiR-200c regulates tumor growth and chemosensitivity to cisplatin in osteosarcoma by targeting AKT2

**DOI:** 10.1038/s41598-017-14088-3

**Published:** 2017-10-19

**Authors:** Yang Liu, Shu-Tao Zhu, Xiao Wang, Jun Deng, Wei-Hua Li, Peng Zhang, Bing-Shan Liu

**Affiliations:** Department of Orthopedic, Huaihe Hospital of Henan University, Baobei Road 8, 475000 Kaifeng, Henan China

## Abstract

MicroRNAs (miRNAs) expression aberration has been discovered in almost all human cancers, thus offering a group of potential diagnostic markers, prognostic factors and therapeutic targets in tumorigenesis. Now our data showed that miR-200c, which is downregulated in osteosarcoma tissues, drives chemosensitivity to cisplatin in osteosarcoma. We demonstrated that AKT2 is a direct target of miR-200c, Spearman’s rank correlation analysis showed that the expression levels of AKT2 and miR-200c in 35 pairs of osteosarcoma specimens were inversely correlated. Moreover, miR-200c inhibited cell proliferation and cell migration. Taken together, for the first time, our results demonstrate that miR-200c plays a significant role in osteosarcoma tumor growth and chemosensitivity by regulating AKT2, which may provide a novel therapeutic strategy for treatment of osteosarcoma.

## Introduction

Osteosarcoma is a highly malignant bone cancer associated with locally aggressive growth and early metastatic potential. The origin and etiology of osteosarcoma is further complicated by its extreme rearranged genome, lack of precursor lesions, and high genetic instability. Intensive chemotherapy combined with aggressive surgical techniques have improved survival; however, patients with metastatic disease or with recurrent disease at time of diagnosis have an extremely poor prognosis, with only 20% surviving at 5 years^1–3^. Thus, it is essential to developing novel and effective diagnostic and therapeutic strategies for osteosarcoma.

MicroRNAs are small noncoding regulatory RNA molecules, with profound impact on a wide array of biological processes. MicroRNAs have been recently implicated in the regulation of tumorigenesis, differentiation, proliferation, and survival through the inhibition of major cellular pathways^4–9^. Among them, miR-200c has been demonstrated to function as a tumor suppressor, and loss of miR-200c expression has been reported in many cancer types, restoration of miR-200c expression has been shown to abrogate tumorigenesis^10–14^. To date, some genes have been identified as miR-200c target genes, including K-RAS, CDK2, ZEB2, Snail1, USP25, HMGB1^15–20^, which are involved in pathogenesis of cancers. A number of reports have investigated the role of miRNAs in osteosarcoma. However, the molecular mechanism of miR-200c repression in osteosarcoma has not been determined.

AKT is a serine/threonine kinase that plays a central role in tumorigenesis. Among the members of AKT family, AKT2, a pro-survival protein, is activated by the phosphatidylinositol 3′ kinase (PI3K) pathway. The activation of the PI3K/AKT pathway is associated with aggressive phenotypes and poor outcomes in human cancers^21^. Activation of the AKT pathway is frequently observed in cancer. Overexpression of AKT2 was frequently discovered in breast cancer and HCC^22,23^. Recent study reported that AKT2 was activated in prostate cancer cells in response to oxidative stress, resulting in enhanced cell migration and survival^24^. AKT2 has also been shown as an independent prognostic marker for the development and progression of HCC^22^. Recent studies indicated that AKT2 could be regulated by miRNAs. MiR-708 targeted AKT2 to inhibit tumor growth of prostate cancer, and miR-203 targeted AKT2 to sensitize colon cancer cells to chemotherapy^25,26^. Thus, AKT2 silencing has become an efficient therapeutic strategy in osteosarcoma, but it is still far from optimal and novel therapeutic strategies are needed urgently.

In the present study, we demonstrated that miR-200c was downregulated in human osteosarcoma. Then, we will ask several important questions in this study: (1) what are the roles of miR-200c in osteosarcoma; (2) what is the potential direct target of miR-200c that may be associated with cancer development; and (3) whether miR-200c overexpression inhibits cell proliferation and migration; (4) What role of miR-200c and underlying mechanisms in osteosarcoma resistance to cisplatin treatment. The answers of these questions would provide new insights into the molecular mechanism of osteosarcoma development as well as provide new therapeutic strategy for osteosarcoma treatment in the future.

## Results

### MiR-200c expression is down-regulated in human osteosarcoma tissues and cell lines

**Figure 1:**
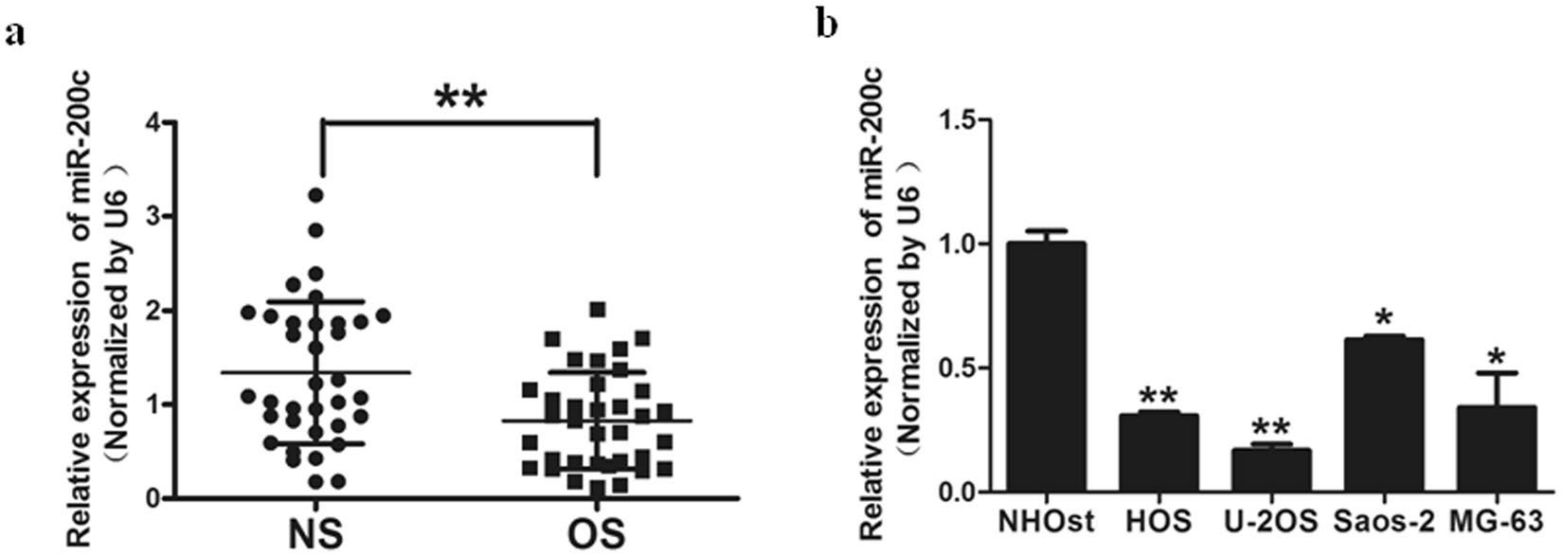
MiR-200c expression was downregulated in human osteosarcoma tissues and cells lines. (**a**) Relative miR-200c expression levels were analyzed by qRT-PCR in 35 pair of osteosarcoma(OS) tissues compared with adjacent non-cancerous tissues(NS). U6 RNA level was used as an internal control. (**b**) Relative miR-200c expression was analyzed in normal osteoblast cells (NHOst) and four osteosarcoma cell lines, HOS, Saos-2, MG-63 and U-2OS. Data represent mean ± SD of 3 replicates. * indicated significant difference at *P* < 0.05; ** indicated significant difference at *P* < 0.01.

To investigate the role of miR-200c in osteosarcoma, we evaluated the expression levels of miR-200c in 35 pairs of normal tissues and osteosarcoma tissues by qRT-PCR (Fig. 1a). The results showed that the expression of miR-200c was consistently lower in the osteosarcoma tissues. In addition, expression of miR-200c in four osteosarcoma cell lines, HOS, Saos-2, MG-63 and U-2OS, was significantly decreased compared with the normal osteoblast cells NHOst (Fig. 1b). Our results firstly indicated that miR-200c was downregulated in osteosarcoma tissues and cell lines.

### MiR-200c inhibits the activity of cell proliferation and cell migration

**Figure 2:**
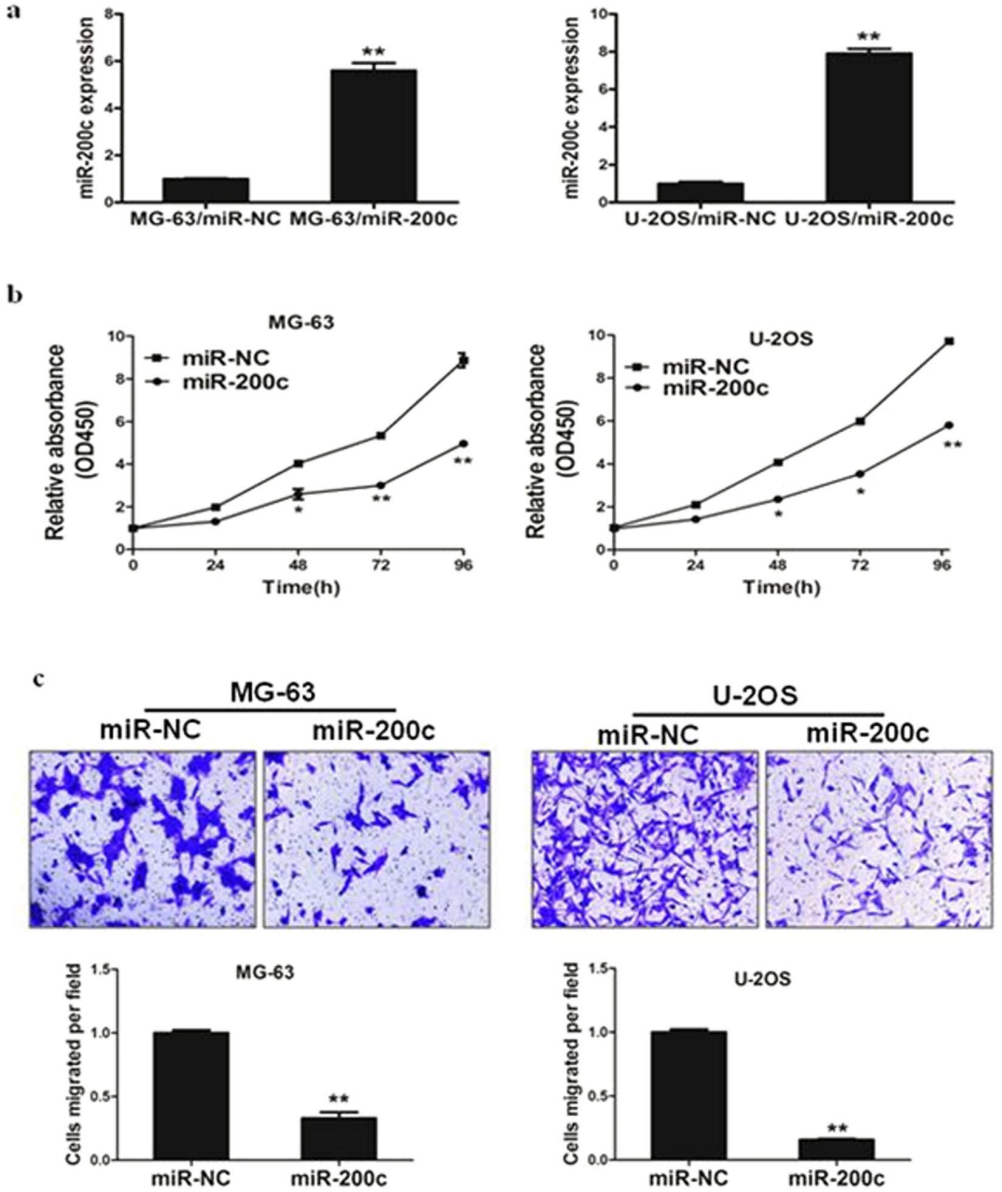
miR-200c inhibits the activity of cell proliferation and cell migration. (**a**) Relative expression levels of miR-200c in MG-63/miR-NC, MG-63/miR-200c, U-2OS/miR-NC and U-2OS/miR-200c stable cell lines were confirmed by RT-qPCR. **(b)** Cells were plated 2000 cells per well in 96-well plates, and cell proliferation was determined using Cell Counting Kit-8 (CCK-8) to detect the absorbance at 450 nm every day. **(c)** Migration assay of stable cells were performed as previously described. Data represent mean ± SD from 3 replicates. * indicated significant difference at *P* < 0.05; ** indicated significant difference at *P* < 0.01.

To examine the role of miR-200c during carcinogenesis of human osteosarcoma, MG-63 and U-2OS cells were infected with lentivirus expressing miR-200c or miR-NC(negative control). After selection by puromycin, stable cell lines termed as MG-63/miR-NC, MG-63/miR-200c, U-2OS/miR-NC and U-2OS/miR-200c were established. RT-PCR analysis demonstrated miR-200c was highly expressed in MG-63/miR-200c and U-2OS/miR-200c cells, confirming that stable cell line over-expressing miR-200c was successfully established (Fig. 2a).

To further study the role of miRNA-200c in regulating cell proliferation and cell migration, we found that cell growth and migration were attenuated in miR-200c cells compared with miR-NC cells (Fig. 2b and c). Thus, our results show that miR-200c is responsible for suppressing cell proliferation and cell migration, thus functioning as a tumor suppressor in osteosarcoma cells.

### AKT2 is a direct target of miR-200c, osteosarcoma tissues exihibits higher levels of AKT2 which are inversely correlated with miR-200c expression

**Figure 3:**
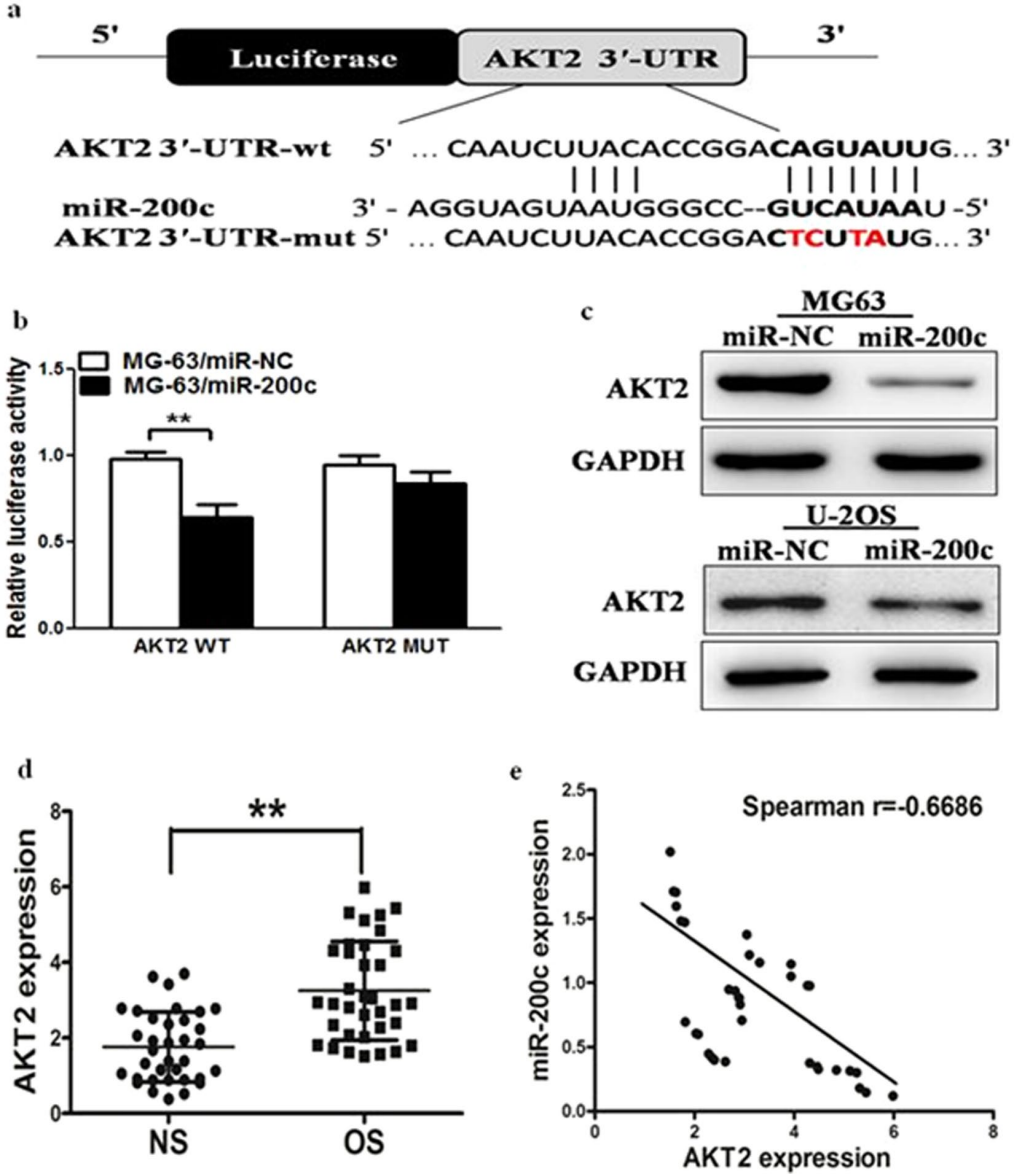
AKT2 is a direct target of miR-200c, osteosarcoma tissues exihibits higher levels of AKT2 which are inversely correlated with miR-200c expression. (**a**) Sequence of the miR-200c-binding site within the human AKT2 3′-UTR and a schematic diagram of the reporter construct showing the entire AKT2 3′-UTR sequence and the mutated AKT2 3′-UTR sequence. The mutated nucleotides of the AKT2 3′-UTR are labeled in red. (**b**) Luciferase assay on MG-63 cells, which were co-transfected with miR-NC or miR-200c and a luciferase reporter containing the full length of AKT2 3′-UTR (WT) or a mutant (Mut) in which four nucleotides of the miR-200c-binding site were mutated. Luciferase activities were measured 24 hours post transfection. MiR-200c markedly suppressed luciferase activity in AKT2 3′-UTR (WT) reporter constructs. The data are means ± SEM. for separate transfections (n = 4). (**c**) The immunoblotting showed that expression levels of AKT2 were decreased in cells with miR-200c overexpression. (**d**) The expression of AKT2 in human normal tissues and osteosarcoma specimens was determined by RT-qPCR, and fold changes were obtained from the ratio of AKT2 to GAPDH levels. (**e**) Spearman’s correlation analysis was used to determine the correlation between the expression levels of AKT2 and miR-200c in human osteosarcoma specimens. Data represent mean ± SD of 3 replicates. ** indicated significant difference at *P* < 0.01.

To fully understand the mechanisms of miR-200c in osteosarcoma, TargetScan search program was used to predict targets of miR-200c, which AKT2 has been thought to be putative target of miR-200c (Fig. 3a). MG-63 cells were cotransfected with the wild (WT) or mutated (Mut) AKT2 luciferase reporter vector together with miR-200c or miR-NC for 24 h, and luciferase activities in those cells were measured. As shown in Fig. 3b, luciferase activities were significantly reduced in those cells transfected with the wild sequence and miR-200c, but not in the cells with the mutant sequence and miR-200c. Then, western blotting analysis was conducted to measure the levels of AKT2 protein, we found that the expression of AKT2 protein was downregulated in miR-200c treated cells. (Fig. 3c). These results suggest that miR-200c directly targets AKT2 by binding its seed region to its 3′-UTRs in osteosarcoma cells. Furthermore, we measured the mRNA levels of AKT2 in osteosarcoma specimens and normal tissues. The results showed that the average expression levels of AKT2 were significantly higher in tumor tissues than those in the normal tissues (Fig. 3d). Then, we determine the correlation between AKT2 levels and miR-200c expression levels in the same osteosarcoma tissues. As shown in Fig. 3e, Spearman’s rank correlation analysis showed that the expression levels of AKT2 and miR-200c in 35 pairs of osteosarcoma specimens were inversely correlated (Spearman’s correlation r = −0.6686).

### Overexpression of miR-200c increases chemosensitivity of osteosarcoma cells to cisplatin by inhibiting its target AKT2

**Figure 4:**
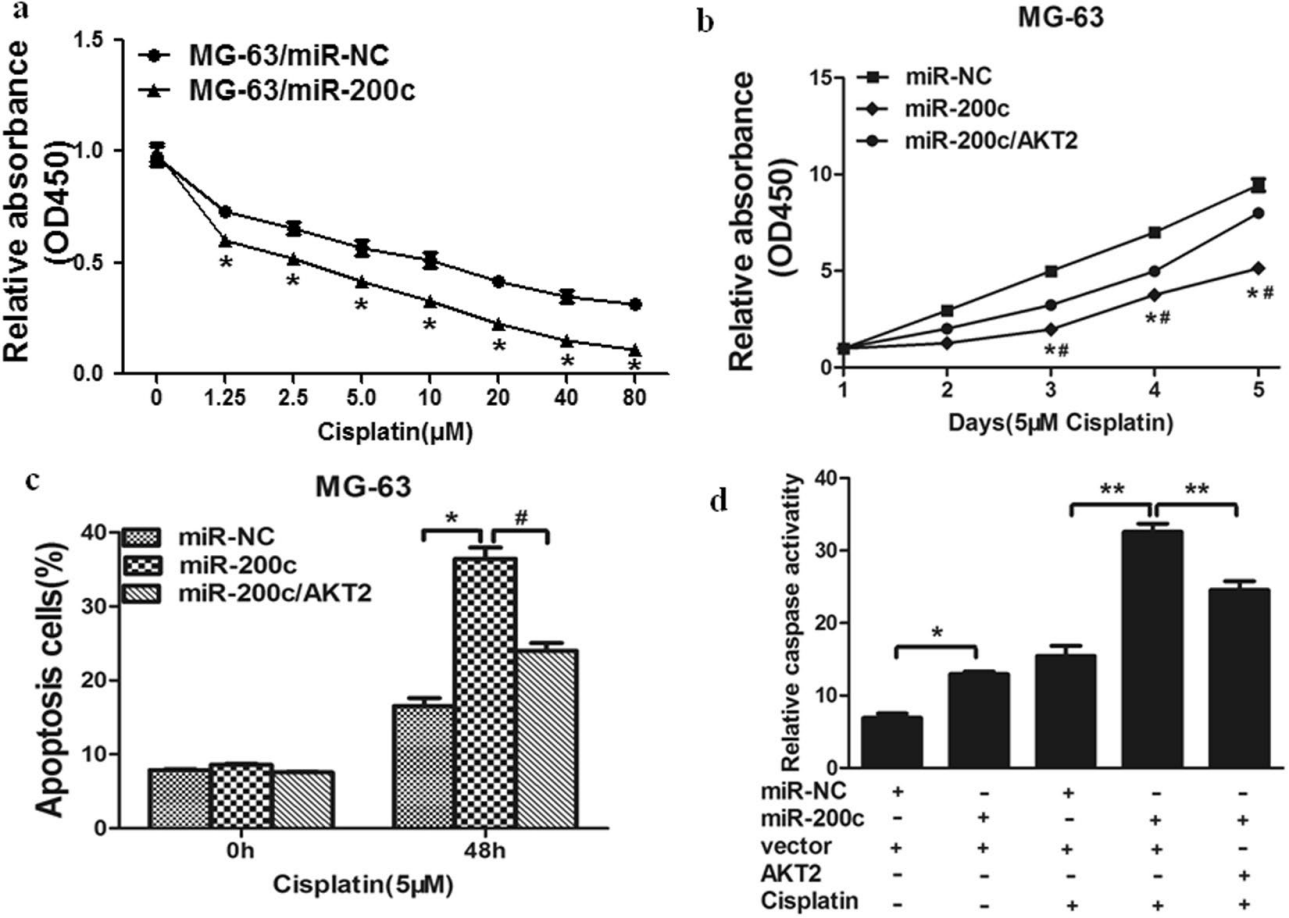
Overexpression of miR-200c increases chemosensitivity of osteosarcoma cells to cisplatin by inhibiting its target AKT2. (**a**) MG-63 cells stably expressing miR-NC or miR-200c were pretreated with cisplatin for indicated concentrations, then subjected to CCK8 Assay. **(b)** MG-63 cells stably expressing miR-NC, miR-200c or miR-200c forced expression of AKT2 were pretreated with 5 μM of cisplatin for indicated time points, then subjected to CCK8 Assay, apoptosis analyse by flow cytometry **(c)** and Caspase3 Assay **(d)**. Data represent mean ± SD of 3 replicates. * or ^#^ indicated *P* < 0.05. ** indicated *P* < 0.01. * indicates significant difference compared to control; ^#^ indicates significant difference compared to miR-200c forced expression of AKT2 treatment.

Resistance to cisplatin treatment is one of the major causes for the failure of chemotherapy in treating osteosarcoma. Therefore, it is important to discover new strategies to increase the effectiveness of cisplatin for therapeutic purposes. Our results showed that overexpression of miR-200c in MG-63 cells significantly increased chemosensitivity to treatment of cisplatin (Fig. 4a). Furthermore, cell growth rate in the presence of cisplatin (5 μM) was assayed by CCK-8 proliferation assay at different time points, forced expression of AKT2 reversed miR-200c-induced osteosarcoma chemosentivity to cisplatin (Fig. 4b). To further study whether miR-200c and its target AKT2 play a role in cell apoptosis in the presence of cisplatin treatment, FACS analysis was performed to detect cell apoptosis rates. The combination of miR-200c and cisplatin treatment significantly induced cell apoptosis, whereas forced expression of AKT2 partially abolished the effect induced by miR-200c plus cisplatin treatment (Fig. 4c). Moreover, we found that compared with miR-200c or cisplatin treatment alone, the activities of caspase-3, a key executor of cell apoptosis, were significantly upregulated upon combination treatment of miR-200c and cisplatin, whereas forced expression of AKT2 attenuated the activation of caspase-3 during the treatment (Fig. 4d). These results indicated that miR-200c renders osteosarcoma cells more sensitive to cisplatin treatment, miR-200c and cisplatin combination induced apoptotic effect through targeting AKT2 in osteosarcoma cells.

### MiR-200c inhibits tumor growth *in vivo*

**Figure 5:**
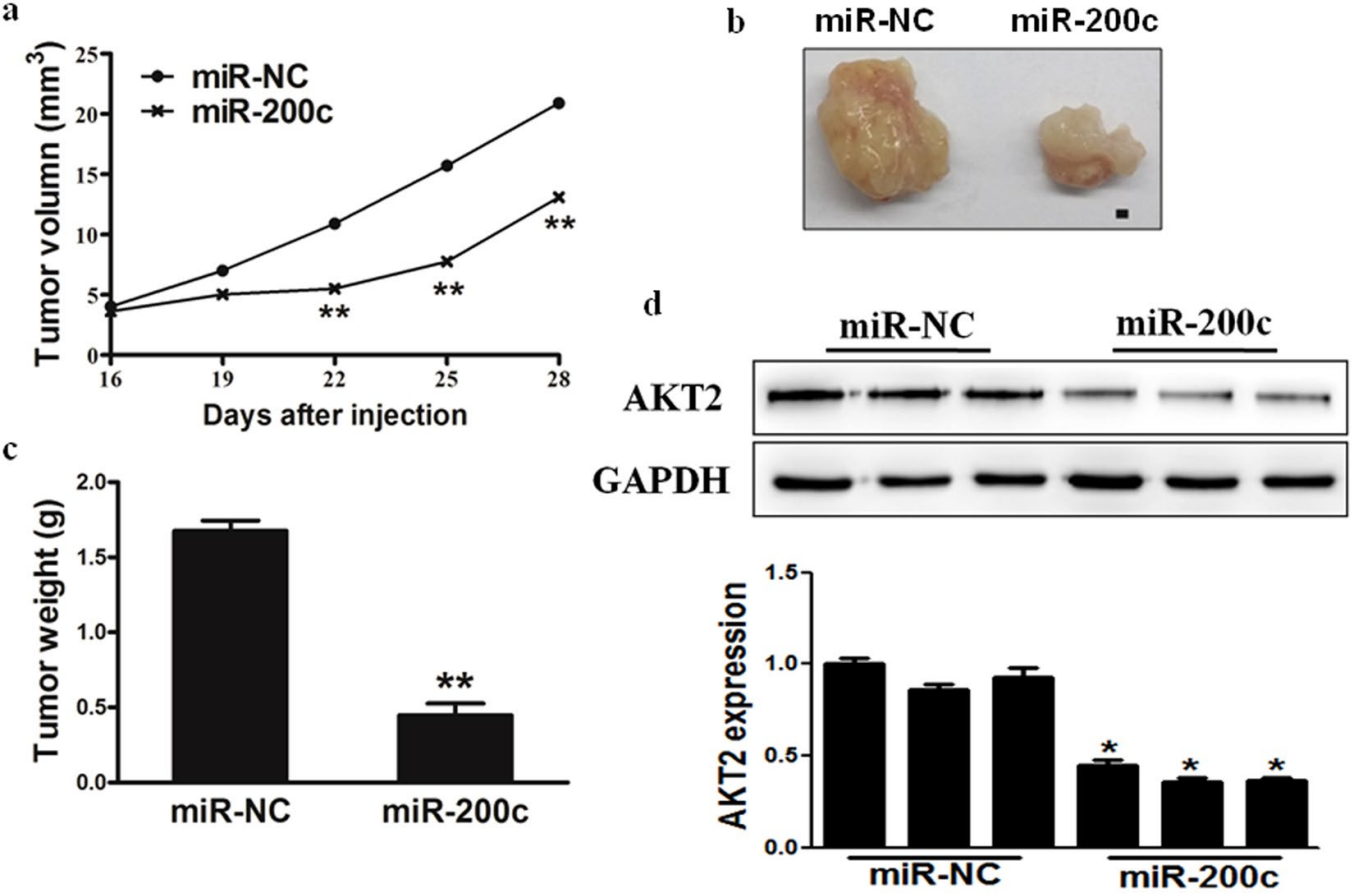
MiR-200c inhibits tumor growth *in vivo*. (**a**,**b**,**c)** Effect of miR-200c on the growth of MG-63 cells inoculated into nude mice. Male BALB/c nude mice were subcutaneously injected with 5 × 10^6^ MG-63 cells infected with lentiviruses harboring miR-NC or miR-200c. Tumor volume and weight were monitored over time as indicated, and the tumor was excised and weighed after 28 days. MiR-200c overexpression causes a decrease in tumor volume and weight. Bar = 1mm. **(d)** The expression levels of AKT2 from the tumor tissues of miR-200c expressing group were lower than that of miR-NC group by immunoblotting assay and qRT-PCR. Data were presented by mean ± SD. * indicated significant difference at *P* < 0.05; ** indicated significant difference at *P* < 0.01.

In order to test whether miR-200c attenuates progression of osteosarcoma *in vivo*, we engineered MG-63 cells to stably express miR-NC or miR-200c, which were subsequently implanted into both posterior flanks of immunodeficient mice, and tumor sizes were measured after 2 weeks. From the 2^nd^ to 4^th^ week, miR-NC-injected group developed significantly larger tumors than miR-200c group (Fig. 5a). MiR-200c stable-expressing cells generated xenografts that were statistically significantly smaller than control (Fig. 5b). Meanwhile, the final tumor weight of miR-NC group was much heavier than miR-200c group (Fig. 5c). In agreement with *in vitro* studies, the levels of AKT2 from the tumor tissues of miR-200c expressing group were lower than that of miR-NC group by immunoblotting assay and qRT-PCR (Fig. 5d). Taken together, these results suggest that miR-200c inhibits tumor growth through targeting AKT2 *in vivo*.

## Discussion

MiRNAs are small non-coding RNAs that post-transcriptionally regulate gene expression. It has suggested that the expression of up to 30% of genes may be affected by miRNAs; thus miRNAs can potentially regulate thousands of genes^27^. MiRNAs act like master regulators of gene expression for many important biological pathways. which can act as oncogenes or tumour suppressors depending on their gene targets, and previous studies have shown that miRNA expression is often dysregulated in several cancers^9^. Recent studies have been reported that miR-200c plays a potential role as a tumor suppressor in many kinds of cancers. However, there are no results referring to the role of miR-200c in osteosarcoma at present. In this study, we found that the expression of miR-200c is downregulated in osteosarcoma samples compared with normal tissues. Moreover, overexpression of miR-200c significantly inhibited osteosarcoma cell proliferation, migration, tumor growth and chemoresistance to cisplatin.

AKT2 play a pivotal role in the transduction of several growth or differentiation factor stimuli. It has been reported that the expression levels of AKT2 are related to the malignant degree of cancers, including osteosarcoma, breast cancer, lung cancer and other cancers^28–33^. Recently, accumulating evidence has indicated that expression levels of the AKT2 family can be regulated by miRNAs. In our study, AKT2 oncogene has been experimentally validated as the novel target of miR-200c. Firstly, luciferase reporter assay confirmed that miR-200c directly recognize the 3′-UTR of AKT2 transcripts. Secondly, AKT2 expression was significantly abolished in osteosarcoma cells which miR-200c stablely-expressed. Finally, osteosarcoma tissues exihibits higher levels of AKT2 which are inversely correlated with miR-200c expression. Taken together, the present study provides the first evidence that miR-200c is significant in suppressing osteosarcoma growth through inhibition of AKT2 translation.

Despite having a better understanding of the molecular events that govern the osteosarcoma than ever before, it remains a clinical challenge in the treatment of osteosarcoma. Several studies have suggested that miRNAs are novel players in the development of chemoresistance. Recent study showed that miR-143 acts as a tumor suppressor by targeting N-RAS and enhances temozolomide-induced apoptosis in glioma, while miR-124 governs glioma growth and angiogenesis and enhances chemosensitivity by targeting R-Ras and N-Ras^34,35^. MiR-200c has been reported to inhibits autophagy and enhances radiosensitivity in breast cancer cells by targeting UBQLN1; meanwhile, miR-200c increases the radiosensitivity of non-small-cell lung cancer cell line A549 by targeting VEGF-VEGFR2 pathway^36,37^. In our study, we found that forced overexpression of miR-200c promoted the effects of cisplatin. Flow cytometer Assay demonstrated that the higher sensitiveness of MG-63 cells with miR-200c to cisplatin was induced by apoptosis. Thus, it is important that a miR-200c restoration approach may offer a new modulation strategy to overcome chemoresistance to cisplatin in osteosarcoma.

In summary, we have identified a link between miR-200c and AKT2 that is a novel constituent of osteosarcoma tumorigenesis. MiR-200c regulate cell proliferation, cell migration and chemosensitivity to cisplatin in osteosarcoma by targeting AKT2. Now despite having a better understanding of the molecular events that govern the osteosarcoma than ever before, it remains a clinical challenge in the treatment of osteosarcoma. Identification of new biomarkers that play a central role in the progression of osteosarcoma will benefit diagnosis and targeting therapy of cancer.

## Materials and Methods

### Human tissue samples

Human osteosarcoma tumor samples were obtained from 35 patients from the Department of orthopedics, Huaihe Hospital of Henan University. The identities of all tumor, normal muscle and bone samples were confirmed by an experienced pathologist. This study was approved by the Research Ethics Committee of Huaihe Hospital of Henan University. Informed consents were obtained from all subjects participating in the study. Tissue samples were collected at surgery, immediately frozen in liquid nitrogen and stored until total RNAs or proteins were extracted.

### Cell culture and reagents

Human osteosarcoma cell lines HOS, U-2OS, Saos-2, MG-63 and normal osteoblast cell NHOst were purchased from the Type Culture Collection of the Chinese Academy of Sciences. Cells were cultured in Dulbecco’s modified Eagle medium (DMEM) supplemented with 10% fetal bovine serum (FBS) and antibiotics (100 units/ml penicillin and 100 mg/ml streptomycin). Cells were incubated at 37 °C in a humidified atmosphere of 5% CO2 in air. Antibodies against AKT2 and GAPDH were from Bioworld Technology (Atlanta, Georgia 30305, USA).

### Lentivirus packaging and stable cell lines

The lentiviral packaging kit was purchased from Open Biosystems (Huntsville, AL, USA). Lentivirus carrying hsa-miR-200c or hsa-miR-negative control (miR-NC) was packaged following the manufacturer’s manual. Lentivirus were packaged in HEK-293T cells and collected from the medium supernatant. Stable cell lines were established by infecting lentivirus into cells and selected by puromycin.

### RNA extraction, reverse transcription PCR and quantitative real time-PCR

RNA was isolated from harvested cells or human tissues with Trizol reagent according to the manufacturer’s instruction (Invitrogen, CA, USA). To measure expression levels of miR-200c, stem-loop specific primer method was used. Expression of U6 was used as an endogenous control. To determine the mRNA levels of AKT2, total RNAs were reversely transcribed by using the RT Reagent Kit (Vazyme, Nanjing, China). Housekeeping gene GAPDH was used as internal control. The cDNAs were amplified by qRT-PCR using SYBR Green Master Mix (Vazyme, Nanjing, China) on a 7900HT system, and fold changes were calculated by relative quantification (2^−△△Ct^).

### Cell proliferation assay

Cells (3,000 cells per well) were seeded onto 96-well plates and incubated in corresponding medium supplemented with 10% FBS. After indicated time incubation, we added CCK-8 into each well, followed by 1–2 hour incubation. Absorbance value at 450 nm was then measured. Experiments were carried out in triplicate.

### Migration assay

Migration assay was determined using 24-well BD Migration chambers (BD Biosciences, Cowley, UK) in accordance with the manufacturer’s instructions. Cells were seeded at 5 × 10^4^ cells/well in the upper well of the migration chamber in DMEM without serum, the lower chamber well contained DMEM supplemented with 10% FBS which aim to stimulate cell migration. After incubation for 16–20 h, noninvading cells were removed from the top well with a cotton swab while the bottom cells were fixed with 3% paraformaldehyde, stained with 0.1% crystal violet, and extracted with 33% acetic acid. Then cells were detected quantitatively using a standard microplate reader (OD at 570 nm). Three independent experiments were conducted in triplicate.

### Immunoblotting

Cells were washed with ice-cold PBS buffer, scraped from the dishes, and centrifuged at 12,000 rpm, 4 °C for 15 min. Cell lysates were prepared using RIPA buffer supplemented with protease inhibitors (100 mM Tris, pH 7.4, 150 mM NaCl, 5 mM EDTA, 1% Triton X-100, 1% deoxycholate acid, 0.1% SDS, 2 mM phenylmethylsulfonyl fluoride, 1 mM sodium orthovanadate, 2 mM DTT, 2 mM leupeptin, 2 mM pepstatin). The supernatants were collected and protein concentration was determined using BCA assay (Beyotime Institute of Biotechnology, Jiangsu, China). Tumor tissues were grinded into powder in liquid nitrogen with RIPA buffer, and the total tissue proteins were extracted as described above. Aliquots of protein lysates were fractionated by SDS-PAGE, transferred to a PVDF membrane (Roche, Switzerland), and subjected to immunoblotting analysis according to the manufacturer’s instruction. ECL Detection System (Thermo Scientific, Rockford, IL, USA) was used for signal detection.

### Luciferase reporter assay

The 3′-UTRs of AKT2 were synthesized and annealed, then inserted into the SacI and HindIII sites of pMIR-reporter luciferase vector (Ambion) at downstream of the stop codon of the gene for luciferase. For its mutagenesis, the sequences complementary to the binding site of miR-200c in the 3′-UTR (AKT2: CAGUAUU) was replaced by CTCUTAU. These constructs were validated by sequencing. MG-63 cells were seeded into a 24-well plate for luciferase assay. After cultured overnight, cells were cotransfected with the wild-type or mutated plasmid, pRL-TK plasmid, and equal amounts of miR-200c or miR-NC. Luciferase assays were performed 24 h after transfection using the Dual Luciferase Reporter Assay System (Promega, WI, USA). Experiments were performed in three independent replicates.

### *In Vitro* Chemosensitivity array

Cells were seeded at a density of 4,000 cells per well in a 96-well plate overnight. Freshly prepared cisplatin (Sigma-Aldrich, St. Louis, MO, USA) was added with the final concentration ranging from 1.25 to 80 μM. 48 h later, cell viability was assayed by CCK8 kit.

### Apoptosis Assay

Apoptosis were measured by flow cytometry. For AnnexinV staining, 5 μL phycoerythrin-Annexin V, 5 μL propidium iodide (BD Pharmingen) and 400 μL 1 × binding buffer were added to the samples, which were incubated for 15 min at room temperature in the dark. Then the samples were analyzed by flow cytometry (FACS Canto II, BD Biosciences) within 1 h. The data were analyzed using FlowJo software. Three experiments were performed in triplicate.

### Caspase-3 Activity Assay

The activity of caspase-3 was determined using the Beyotime caspase-3 activity kit. Cell lysates were prepared and incubated with reaction buffer containing caspase-3 substrate (Ac-DEVD-pNA) after the treatment as indicated. Caspase-3 activity assay were performed on 96-well plates by incubating 10 μL protein of cell lysate per sample in 80 μL reaction buffer containing 10 μL caspase-3 substrate(Ac-DEVD-pNA; 2 mM) at 37 °C for 2 h according to the manufacturer’s protocol. The reaction was then measured at 405 nm for absorbance.

### Tumorigenesis in nude mice

Male BALB/c nude mice (6-weeks-old) were purchased from Shanghai Laboratory Animal Center (Chinese Academy of Sciences, Shanghai, China) and maintained in special pathogen-free (SPF) condition for one week. Animal handling and experimental procedures were in accordance with the Guide for the Care and Use of Laboratory Animals. MG-63 cells stably expressing miR-200c or miR-NC were injected subcutaneously into both flanks of nude mice (5 × 10^6^ cells in 100 μl). Tumor sizes were measured using vernier caliper every two days when the tumors were apparently seen and tumor volume was calculated according to the formula: volume = 0.5 × Length × Width^2^. 24 days after implantation, mice were sacrificed and tumors were dissected. Total proteins and RNAs were extracted for immunoblotting and qRT-PCR.

### Ethics statement

All animal experiments were approved by the Committee of Laboratory Animal Experimentation of Henan University. All methods were performed in accordance with the relevant guidelines and regulations. The mice used in this study were housed in a controlled specific pathogen-free (SPF) environment and cared according to the approved protocol.

### Statistical analysis

All experiments were performed three times and data were analyzed with GraphPad Prism 5(La Jolla, CA, USA). The correlation between miR-200c expression and AKT2 levels in osteosarcoma tissues were analyzed using Spearman’s rank test. Statistical evaluation for data analysis was determined by *t*-test. The differences were considered to be statistically significant at *P* < 0.05.
